# MRI Features of Diffuse Glioneuronal Tumor with Oligodendroglioma-like features and Nuclear Clusters: A Case Report and Literature Review

**DOI:** 10.2174/0115734056482743260824063132

**Published:** 2026-09-11

**Authors:** Dabiao Deng, Chongzhu Fan, Yanying Yang, Wensheng Wang, Huafei Zhao, Shougui Gao

**Affiliations:** 1 Department of Imaging, Guangdong Sanjiu Brain Hospital, Guangzhou 510510, Guangdong Province, China;; 2 Department of Pathology, Guangdong Sanjiu Brain Hospital, Guangzhou 510510, Guangdong Province, China;; 3 Department of Oncology, Guangdong Sanjiu Brain Hospital, Guangzhou 510510, Guangdong Province, China

**Keywords:** Diffuse glioneuronal tumor, Nuclear clusters, Brain tumor, Magnetic resonance imaging, Diffusion-weighted imaging, Prognosis

## Abstract

**Introduction:**

Accurate diagnosis of diffuse glioneuronal tumors with oligodendroglioma-like features and nuclear clusters (DGONC) remains challenging due to scarce clinical and imaging evidence. Further systematic studies are therefore needed to characterize this tumor entity.

**Case Presentation:**

A 7-year-old boy presented with a 3-day history of headache and vomiting. Cranial Magnetic Resonance Imaging (MRI) revealed a well-circumscribed mass in the right temporal lobe with a ground-glass appearance and no contrast enhancement or restricted diffusion. After gross total resection, histopathology showed diffusely infiltrating oligodendroglioma-like cells accompanied by “pennies on a plate” multinucleated cells and nuclear clusters. DNA methylation profiling confirmed the diagnosis of DGONC (calibrated score, 0.99), and chromosomal copy number analysis demonstrated multiple chromosomal gains without monosomy 14. The patient received radiotherapy alone postoperatively and remained clinically stable at the 35.5-month follow-up. This case suggests that the discordance between high-grade histology and an indolent, low-grade radiological appearance is an important clinical feature of DGONC.

**Conclusion:**

This case demonstrates that DGONC can present with non-epileptic onset and without monosomy 14. When a brain tumor exhibits characteristic histology yet a low-grade radiological appearance, DGONC should be considered and the diagnosis confirmed by methylation analysis. Treatment decisions must be made cautiously, based on the extent of surgical resection and histological features.

## 1 INTRODUCTION

The classification of Central Nervous System (CNS) tumors has evolved markedly since the 2016 integration of histopathology with molecular features. Emerging entities continue to surface, though their neuroradiological profiles often remain poorly characterized. Clarifying these imaging signatures has become a priority to sharpen diagnostic precision. Among these newly codified tumors is the DGONC, added as a provisional entity in the 2021 WHO classification [1,2]. At present, understanding of DGONC rests on scattered case reports and limited case series, rendering it an unfamiliar diagnosis for many clinicians and radiologists. Therefore, this study aims to describe the MRI findings and clinical features of a 7-year-old boy diagnosed with DGONC, complemented by a literature review, to enhance understanding of this emerging tumor entity.

## 2 CASE DESCRIPTION

A 7-year-old boy was admitted on September 19, 2022, with a chief complaint of headache and vomiting lasting more than 3 days. He had no family history of hereditary disease, and both physical examination and laboratory tests were unremarkable. MRI demonstrated a well-defined lesion in the right temporal lobe. On axial images, the tumor was uniformly hypointense relative to cortex on T1-weighted imaging (T1WI), and hyperintense with ground-glass appearance on T2-weighted imaging (T2WI) and T2-weighted fluid-attenuated inversion recovery (T2-FLAIR), without necrosis, cyst, calcification, or hemorrhage. On diffusion-weighted imaging (DWI) acquired with b = 1000 s/mm^2^, it showed slightly higher signal than cortex, with mildly increased apparent diffusion coefficient (ADC) (tumor: 0.927 × 10^−3^ mm^2^/s; contralateral normal white matter: 0.811 × 10^−3^ mm^2^/s) (Fig. **1**). Our initial imaging diagnosis was a pediatric diffuse glioma. After detailed discussion with the patient’s family, gross total resection of the right temporal lobe lesion was performed. Intraoperatively, the tumor showed soft, ill-defined, grayish-white, and moderate vascularity. Histopathology revealed diffusely infiltrating cells with moderate to marked hypercellularity. Most nuclei were round with perinuclear halos, mimicking oligodendroglioma-like morphology; some cells showed nuclear pleomorphism. Scattered multinucleated tumor cells displayed the classic “pennies on a plate” appearance, together with nuclear clusters. Mitotic figures were conspicuous in hot spots. Necrosis and microvascular proliferation were absent. Immunohistochemistry yielded the following results: Syn (+), Olig 2 (+), GFAP (−), NeuN (+), ATRX (+, retained), P53 approximately 3% (+), IDH 1 (−), BRAF (−), CD 34 (−), EMA (−), H3 K27M (−), ALK (−), H3.3 G34R (−), H3.3 G34V (−), H3 K27me3 (−/+), Met (−), and Ki-67 approximately 15% (+) (Fig. **2**). PCR analysis revealed wild-type BRAF V600E, IDH1, or IDH2. Histopathology suggested a pediatric diffuse glioma, CNS WHO grade 2-3, and the differential diagnosis included a pediatric diffuse glioma with distinct genetic alterations or a glioneuronal tumor. The patient recovered uneventfully after surgery. After discharge, the patient received radiotherapy at another hospital; the specific radiation dose, fractionation schedule, and treatment plan were unknown. The patient did not return to our hospital for follow-up brain MRI. Meanwhile, the specimen underwent molecular profiling at Guangzhou Huayin Medical Laboratory Center, followed by DNA methylation array (EPIC v12.8, brain tumor classifier) at Heidelberg University Hospital. The methylation profile showed a 0.99 calibration score for DGONC (**Supplementary material 1**). Chromosomal copy number variation analysis detected gains in chromosomes 1, 2, 8, 12, 13, and 18, with no definitive monosomy 14 (**Supplementary material 2**). At the 35.5-month telephone follow-up, the family reported that the patient remained stable but did not provide follow-up imaging data (**Supplementary material 3**).

## 3 DISCUSSION

DGONC is a provisional entity in the 2021 WHO Classification of CNS Tumors, characterized by a distinct DNA methylation profile, nuclear cluster morphology, and monosomy 14, without an assigned WHO grade [1-3]. This tumor predominantly occurs in children, and seizures are the most common initial clinical presentation [4-6]. To systematically characterize its clinical and imaging features, we searched PubMed, Scopus, Web of Science, Embase, and the Cochrane databases in May 2026 using the keywords “DGONC” and “(diffuse glioneuronal tumor) AND (oligodendroglioma) AND (nuclear clusters)” in titles and abstracts. This search yielded 17 articles. Inclusion criteria comprised DGONC cases confirmed by histopathological and/or molecular testing with extractable data. After excluding duplicate reports and cases with insufficient information, 12 articles (52 cases) remained (**Supplementary material 4**) [3-14]. Together with one case from our institution, 53 cases were included in the analysis. Of the 53 cases, 25 (47.2%) were male, 21 (39.6%) were female, and sex was not reported in 7. Age was available for 45 cases: range 2–75 years, median 11.0 years; 36/45 cases (80.0%) were ≤18 years. Sixteen cases had documented clinical manifestations. Of these, 11 (68.8%) presented with seizures as the initial symptom. The remaining presented with signs of increased intracranial pressure such as headache and vomiting or focal neurological deficits. In contrast, our case presented with headache and vomiting and remained seizure-free throughout the disease course. This discrepancy suggests that non-epileptic onset of DGONC is not rare, and differential diagnostic considerations should be broadened in clinical practice.

The nomenclature of DGONC reflects its histological hallmarks: diffuse growth, oligodendroglioma-like cells, and nuclear clusters. First systematically described by Deng *et al*. [3], nuclear clusters comprise tightly aggregated nuclei with scant intervening cytoplasm, resembling “pennies on a plate”. No quantitative threshold has been established, and criteria distinguishing nuclear clusters from perivascular pseudo-rosettes or “pennies on a plate”-like multinucleated giant cells remain undefined; their identification relies on nuclear atypia and diffuse, non-perivascular distribution within the tumor parenchyma. Nuclear cluster frequency varies, and clusters may be sparse or focal in confirmed cases, indicating a supportive but not definitive diagnostic feature [3,7]. Immunohistochemistry in the present case showed an Olig 2-positive/Syn-positive/NeuN-positive/GFAP-negative; MAP 2 was not tested. In our aggregated series, Olig 2, Syn, and NeuN were positive in 10 of 11 (90.9%), 19 of 21 (90.5%), and 14 of 15 (93.3%) cases, respectively; MAP 2 was positive in all 12 cases, and GFAP was negative in 13 of 19 (68.4%) cases. These findings reflect the dual glioneuronal differentiation of DGONC, with an Olig 2-positive/Syn-positive/GFAP-negative as its characteristic immunophenotype. This immunophenotype is not unique to DGONC, as Syn and Olig 2 co-expression also occurs in glioneuronal tumors, CNS neuroblastomas, and PNETs, and its morphology closely resembles high-grade gliomas and anaplastic oligodendrogliomas, both features that explain its frequent misdiagnosis prior to recognition as a distinct entity. In this case, the absence of monosomy 14, a common molecular hallmark, introduced diagnostic uncertainty despite immunohistochemical and morphological findings consistent with DGONC; definitive diagnosis was confirmed by DNA methylation profiling (classifier score 0.99). Across 53 aggregated cases, 49 (92.5%) cases were confirmed by methylation profiling, and the remaining 4 cases by integrated histopathological, immunophenotypic, and molecular assessment. DNA methylation profiling thus plays a central role in DGONC diagnosis, whereas immunohistochemistry, despite diagnostic utility, remains insufficient to establish the diagnosis independently.

Monosomy 14 is the most common copy number variation in DGONC, accounting for approximately 90% of cases with available testing in our series. Its biological and clinical significance, however, remains undetermined. Chromosome 14q harbors approximately 1,100 protein-coding genes, inclu-ding several candidate tumor suppressor genes (*e.g*., AKT1, MEG3, DLEU1). Its deletion may contribute to DGONC tumorigenesis, but the specific target gene has not been identified; current inferences are largely drawn from studies on meningioma and colorectal cancer and have not been validated in neuroepithelial tumors [3,8]. Approximately 10% of methylation-confirmed DGONCs, including the present case, lack monosomy 14, suggesting that it is a frequent event rather than an obligatory driver. In other tumor types, 14q deletion is often associated with aggressive behavior, yet in DGONC it coexists with an indolent clinical course. This paradox currently lacks a satisfactory explanation, and no evidence links it to imaging or histological grade. Moreover, four cases in our series were diagnosed as DGONC based solely on histomorphology, immunohistochemistry, and the exclusion of key molecular markers. Therefore, the current value of monosomy 14 lies primarily in diagnostic support rather than in prognostication or treatment decisions; clinically, DGONC should not be readily excluded solely because of the absence of this alteration, especially when the diagnosis is already confirmed by methylation profiling. In addition to monosomy 14, various other chromosomal copy number variations were observed, most commonly 17q gain (23/38, 60.5%), 1q gain (19/36, 52.8%), and 19q deletion (14/42, 33.3%). The present case lacked monosomy 14 and exhibited only multiple chromosomal gains without deletions, a pattern clearly distinct from the common profile (*e.g*., 17q gain and 19q deletion). This rare “no monosomy 14, gains only” feature suggests a possible molecular subtype characterized by multiple chromosomal gains, the clinical significance of which warrants further study. Our series demonstrates a highly heterogeneous mutational landscape in DGONC. Somatic mutations were detected in approximately half of the cases (12/22, 54.5%), involving pathways such as PI3K and RTK/RAS, but no recurrent high-frequency mutations were found. Moreover, IDH1/2, BRAF V600E, and 1p/19q codeletion were consistently negative, indicating that this tumor is independent of the common driver events known in gliomas.

We summarized the imaging features of 21 DGONC cases in our series (**Supplementary material 5**). The tumor predominantly involved the supratentorial superficial region, most frequently the temporal lobe (11/21, 52.4%) and frontal lobe (9/21, 42.9%). On MRI, the lesions typically appeared hypointense on T1WI and hyperintense on T2WI/T2 FLAIR. The solid component showed homogeneous signal in 18 of 19 cases (94.7%), and the tumor margins were well-defined in most cases (16/19, 84.2%). The characteristic “ground-glass appearance” of DGONC was systematically described by Benesch *et al*. [5] as homogeneous, well-circumscribed, high-to-intermediate signal intensity on T2WI and FLAIR relative to the cortex. This term lacks standardized quantification, and not all tumors exhibit this sign; 15 of 19 cases (78.9%) showed the typical appearance. The tumors showed no T2-FLAIR mismatch sign. Small cystic changes or necrosis were common (12/19, 63.2%), peritumoral edema was minimal or absent (15/20, 75.0%), hemorrhage was rare (2/20, 10%), and calcification was present in approximately 40% of cases (7/18, 38.9%). The majority of cases (11/15, 73.3%) showed no restricted diffusion on DWI; however, one case exhibited marked diffusion restriction and was misdiagnosed as an epidermoid cyst [12]. Post-contrast T1WI was predominantly absent (12/20, 60.0%). In 8 of 18 (44.4%) superficial lesions, adjacent calvarial bone remodeling was observed, indicating slow growth. One case exhibited low perfusion on PWI, differing from the typical hyperperfusion of high-grade gliomas; this finding awaits validation in larger cohorts [11]. Overall, the imaging features of DGONC resemble those of a low-grade tumor and markedly diverge from the high-grade histology often observed. This “high-grade pathology, low-grade imaging” dissociation is an important clinical feature distinguishing DGONC from other gliomas and glioneuronal tumors, and was also evident in our case. Additionally, some cases exhibit atypical imaging features. Maurice *et al*. [13] reported a case with concurrent intracranial and sacral involvement, and Torres *et al*. [12] reported a case with recurrence and ventricular dissemination, suggesting that DGONC may have the potential for multifocal growth or cerebrospinal fluid dissemination, and that comprehensive neuraxis screening should be considered in suspected cases. A case of malignant progression following surgery for tumor hemorrhage reported by Xu *et al*. [10] and a case of DGONC with concomitant adrenal ganglioneuroma reported by Shahbandi *et al*. [9] have further broadened the understanding of the clinical spectrum of this tumor. Since PWI, APT, SWI, and other functional MRI were not performed in the present case, the functional imaging assessment has certain limitations. For imaging differential diagnosis, DGONC should mainly be distinguished from ganglioglioma, dysembryoplastic neuroepithelial tumor, and pediatric-type diffuse astrocytoma [15], with key points summarized in Table **1**. Most of these mimics belong to the spectrum of long-term epilepsy-associated tumors, which typically present with chronic seizures [16].

Treatment modalities for DGONC showed marked heterogeneity in our series, with surgery as the cornerstone and diverse adjuvant regimens. Radiotherapy and/or chemotherapy were administered in 68.2% (15/22) of cases. Among them, two patients were managed with a “watch and wait” strategy: one experienced progression after 3.5 months of preoperative observation, and the other progressed 1 year after surgery; both subsequently received active treatment. Surgery alone accounted for 31.8% (7/22) of cases, of whom only one was followed for more than 2 years (108 months); three were followed for less than 2 years, two lacked follow-up data, and one died 1.8 months postoperatively. These observations indicate that the long-term safety of “watch and wait” remains unverified. Currently, no standardized guidelines exist for DGONC, and the comparative efficacy of different strategies has not been clearly established. Regarding prognosis, the literature reports a 5-year overall survival rate of 86%–100%, which is generally favorable [3,4]. In our series, the progression rate was 21.2% (7/33), the mortality rate was 9.4% (3/32), median progression-free survival was 22.5 months, and median overall survival was 27 months. Long-term (≥60 months) progression-free survivors accounted for 29.6% (8/27), with the longest reaching 154 months [7]. The time to progression varied widely (0.8–26 months). Adults and patients with initially diagnosed high-grade tumors showed more rapid progression and worse prognosis, whereas children and patients with gross total resection often followed an indolent course. The prognosis of DGONC may be more favorable than that of conventional high-grade gliomas, but individual variation is pronounced, and reliable molecular or clinical markers to predict the disease course are lacking. In our case, the patient remained progression-free for 35.5 months after gross total resection and radiotherapy alone, consistent with the long-term stable cases reported in the literature. However, radiotherapy constitutes a confounding factor, and the absence of complete postoperative imaging and precise radiotherapy parameters precludes an accurate assessment of the actual contribution of treatment to this stability. This limitation is also shared by the existing literature, which largely consists of retrospective small cohorts with non-uniform treatment protocols and variable follow-up, making it difficult to establish a causal relationship between treatment and outcome. Based on these limitations, we suggest that the management of DGONC should follow an individualized approach. For young patients with gross total resection and no high-risk features, close observation is an option, provided the risk of progression is fully communicated. For those with incomplete resection or high-risk features, adjuvant therapy should be actively considered. Accurate methylation-based diagnosis and standardized follow-up are prerequisites for decision-making. Multicenter prospective studies are needed to identify risk stratification markers, compare the efficacy of different strategies, and provide evidence for treatment decisions.

## CONCLUSION

A case of DGONC presenting with headache and vomiting without seizures is reported. Imaging demonstrated the typical “high-grade pathology–low-grade imaging” discordance. Molecular testing revealed widespread chromosomal gains without monosomy 14. The patient had a favorable outcome after gross total resection and radiotherapy. This case exhibits rare variant features of DGONC in both clinical presentation and molecular profile, further expanding the clinicopathological spectrum of this tumor entity. For DGONC cases with atypical clinical presentation and no monosomy 14, DNA methylation profiling remains the cornerstone of definitive diagnosis. The clinical management of DGONC should follow an individualized approach. Future prospective data are needed to enable risk stratification and develop evidence-based treatment strategies.

## Figures and Tables

**Fig. (1):**
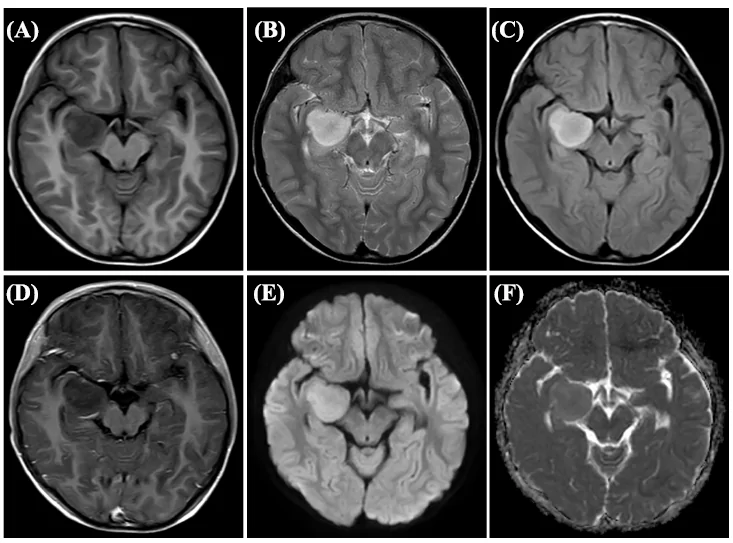
MRI features of the right temporal DGONC. (**A**) Axial T1WI shows homogeneous low signal intensity. (**B**) Axial T2WI and (**C**) T2-FLAIR reveal homogeneous high signal intensity with a ground-glass appearance. (**D**) Axial contrast-enhanced T1WI shows no enhancement. (**E**) Axial DWI (b = 1000 s/mm^2^) shows slightly increased signal relative to cortex, and (**F**) ADC map demonstrates mildly increased signal, with tumor ADC = 0.927 × 10^−3^ mm^2^/s and contralateral normal ADC = 0.811 × 10^−3^ mm^2^/s. No necrosis, cystic change, calcification, or hemorrhage is identified.

**Fig. (2):**
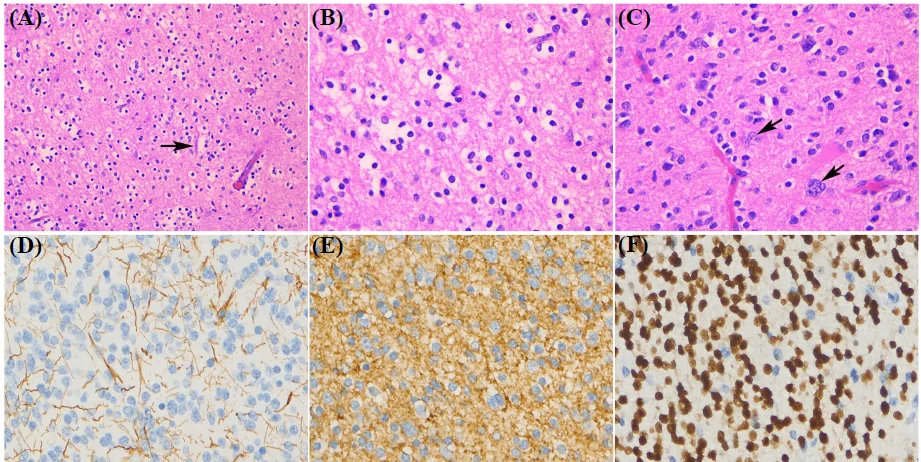
Histopathology of the right temporal DGONC. (**A**, **B**) Diffusely infiltrating tumor cells with round or oval nuclei, translucent cytoplasm, perinuclear halos, and slender interstitial vasculature (A: black arrow, 200×; B: 400×). (**C**) Scattered nuclear clusters formed by multiple tumor cell nuclei within the tumor (black arrow, 400×). (**D**) Negative staining for GFAP (400×). (**E**) Diffuse positivity for synaptophysin (400×). (**F**) Diffuse nuclear staining for OLIG-2 (400×). Notes: Formalin-fixed, paraffin-embedded (FFPE) tissue was sectioned at 4 μm and mounted on precoated glass slides (Thermo Scientific Superfrost Plus, USA). Hematoxylin-and-eosin staining, as well as immunostaining for GFAP, Syn, and OLIG-2 (GeneTex, China), was performed using an OptiView DAB kit (Immunologic, Roche, USA) on a Ventana BenchMark XT immunostainer (Ventana Medical Systems, Tucson, AZ, USA) as previously described.

**Table 1:** Imaging features distinguishing DGONC from pediatric diffuse glioma, ganglioglioma, and DNET.

**Feature**	**DGONC**	**Pediatric Diffuse Glioma**	**Ganglioglioma**	**DNET**
Predominant age	Children (median 9.5 years)	Children	Children/young adults	Children/young adults
Common location	Temporal > frontal (supratentorial)	Supratentorial or infratentorial	Temporal lobe (most common)	Temporal lobe
Imaging morphology	Predominantly solid	Solid or cystic-solid	Cystic with mural nodule	Multicystic, “bubbly” appearance
Margins	Well-defined	Often ill-defined	Well-defined	Well-defined
FLAIR signal	Hyperintense, “ground-glass”	Hyperintense	Hyperintense	Hyperintense rim, wedge-shaped
T2-FLAIR mismatch sign	Absent	Present in some	Absent	Absent
Edema	None or minimal	Prominent (high-grade)	Minimal	None or minimal
Enhancement	Usually absent	Variable (none to marked)	Mural nodule enhancement	Usually absent
DWI	Usually no restricted diffusion	Usually no restricted diffusion; possible in high-grade	Usually no restricted diffusion	Usually no restricted diffusion
PWI	Low perfusion	Low to high perfusion	Low perfusion	Low perfusion

## Data Availability

The data and supportive information is available within the article.

## LIST OF ABBREVIATIONS

MRI: Magnetic resonance imaging
DGONC: Diffuse glioneuronal tumor with oligodendroglioma-like features and nuclear clusters
DNA: Deoxyribonucleic acid
CNS: Central nervous system
WHO: World Health Organization
T1WI: T1-weighted imaging
T2WI: T2-weighted imaging
T2-FLAIR: T2-weighted fluid-attenuated inversion recovery (FLAIR)
DWI: Diffusion-weighted imaging
ADC: Apparent diffusion coefficient
Syn: Synaptophysin
OLIG-2: Oligodendrocyte transcription factor 2
GFAP: Glial fibrillary acidic protein
NeuN: Neuronal nuclei
ATRX: Alpha-thalassemia/mental retardation syndrome X-linked
P53: Tumor protein p53
IDH-1/IDH1/2: Isocitrate dehydrogenase 1/2
BRAF: B-Raf proto-oncogene, serine/threonine kinase
CD 34: Cluster of differentiation 34
EMA: Epithelial membrane antigen
H3 K27M: Histone H3 lysine 27-to-methionine mutation
ALK: Anaplastic lymphoma kinase
H3.3 G34R/G34V: Histone H3.3 glycine 34-to-arginine/valine mutation
H3 K27me3: Histone H3 lysine 27 trimethylation
Met (c-MET): MET proto-oncogene (hepatocyte growth factor receptor)
Ki-67: Marker of proliferation Ki-67
PCR: Polymerase chain reaction
EPIC: Infinium MethylationEPIC BeadChip array (Illumina)
DNET: Dysembryoplastic neuroepithelial tumor
PWI: Perfusion-weighted imaging
MAP 2: Microtubule-associated protein 2
PNET: Primitive neuroectodermal tumor
AKT1: AKT serine/threonine kinase 1
MEG 3: Maternally expressed 3
DLEU 1: Deleted in lymphocytic leukemia 1
PI3K: Phosphatidylinositol 3-kinase
RTK/RAS: Receptor tyrosine kinase/rat sarcoma signaling pathway
APT: Amide proton transfer (imaging)
SWI: Susceptibility-weighted imaging

## STANDARDS OF REPORTING

CARE guidelines were followed.
