# Supplementary material for: MRI Features of Diffuse Glioneuronal Tumor with Oligodendroglioma-like features and Nuclear Clusters: A Case Report and Literature Review
Source: Curr Med Imaging. 2026 Sep 11;22:e15734056482743. doi: 10.2174/0115734056482743260824063132 (PMC13741123; doi:10.2174/0115734056482743260824063132)
Supplement: Supplementary file 1 [file CMIM-22-E15734056482743_SD1.pdf]

1875-6603/26

Send Orders for Reprints to reprints@benthamscience.net

BENTHAM SCIENCE

CrossMark

Current Medical Imaging

Content list available at: https://benthamscience.com/journals/cmir

Supplementary Material

MRI Features of Diffuse Glioneuronal Tumor with Oligodendroglioma-like Features and Nuclear Clusters: A Case Report and Literature Review

Dabiao Deng[1,*], Chongzhu Fan[2,#], Yanying Yang[3,#], Wensheng Wang[1], Huafei Zhao[1] and Shougui Gao[1]

[1]*Department of Imaging, Guangdong Sanjiu Brain Hospital, Guangzhou 510510, Guangdong Province, China*
[2]*Department of Pathology, Guangdong Sanjiu Brain Hospital, Guangzhou 510510, Guangdong Province, China*
[3]*Department of Oncology, Guangdong Sanjiu Brain Hospital, Guangzhou 510510, Guangdong Province, China*

Version 12.8 of the brain classifier results (12.8)

Methylation classes<br>(Highest level >= 0.3, lower levels >= 0.1, all of lowest level)	Calibrated score	Interpretation	
Low Grade Glial/glioneuronal/neuroepithelial Tumours	0.99	match	✓
Low Grade Glioneuronal Tumour	0.99	match	✓
Diffuse Glioneuronal Tumour With Oligodendroglioma Like Features And Nuclear Clusters	0.99	match	✓
Mc Diffuse Glioneuronal Tumour With Oligodendroglioma Like Features And Nuclear Clusters (dgonc)	0.99	match	✓

Legend: ✓ Match (score >= 0.9) ✗ No match (score < 0.9): possibly still relevant for low tumor content and low DNA quality cases.

Class descriptions

MC Diffuse glioneuronal tumour with oligodendroglioma-like features and nuclear clusters (DGONC): The "mc diffuse glioneuronal tumour with oligodendroglioma-like features and nuclear clusters (DGONC)" comprises tumours morphologically characterized by diffuse growth, frequently clear cell histology, and formation of clusters of nuclei, as described in PMID31867747 and 33325069. These tumours virtually all harbour monosomy of chromosome 14. The median age is 9 years, but cases up to an age of 75 years are reported..

Supplementary Figure 1. Methylation-based classification results using Brain Classifier v12.8. The tumor sample was classified as "DGONC" with a calibrated score of 0.99. All hierarchical parent classes showed consistent matching results (scores = 0.99).

DOI: 10.2174/0115734056462689260813104254, 2026, *22*, e15734056482743

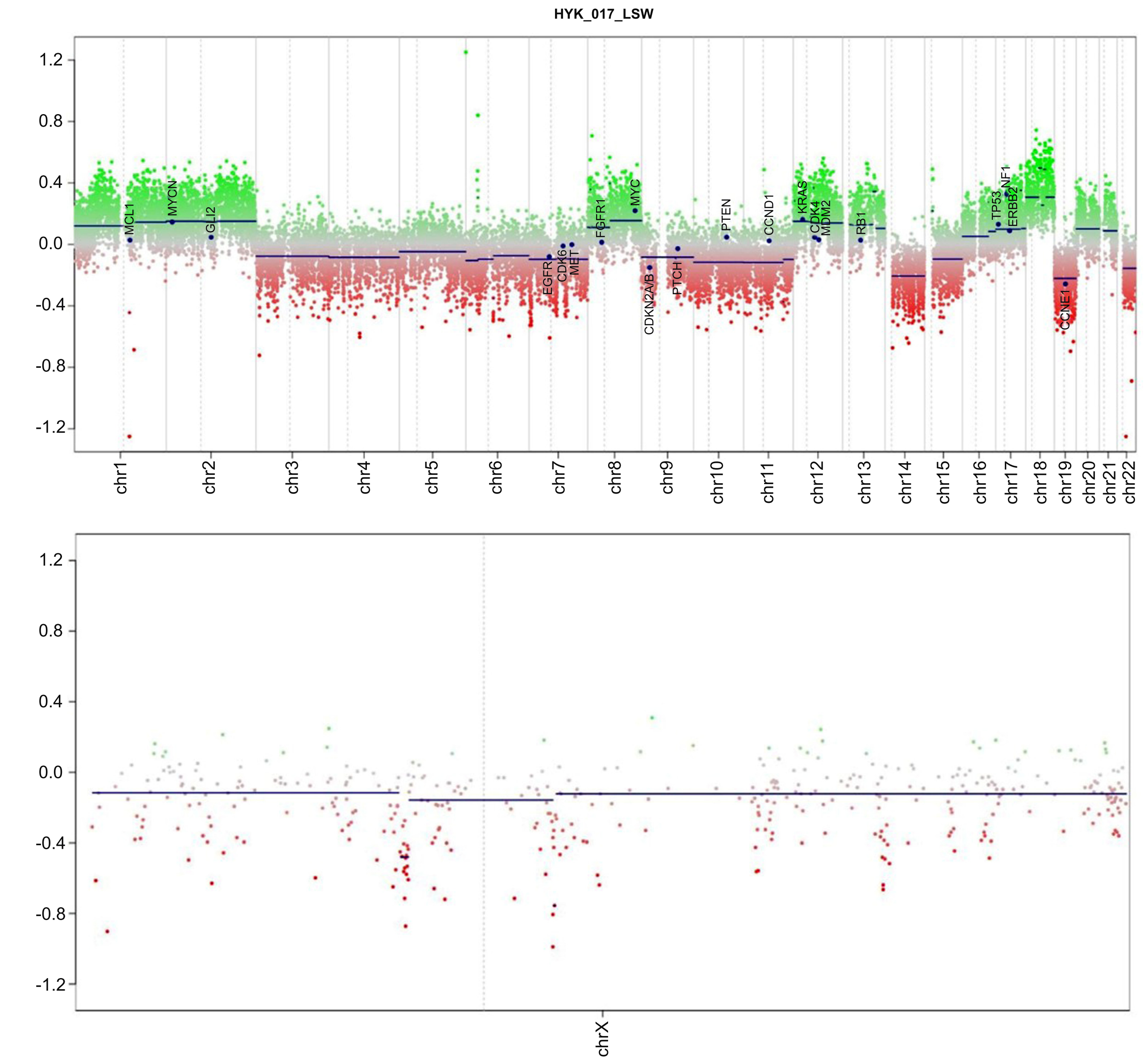

Supplementary Figure 2. Chromosomal copy number variation analysis. The analysis identified chromosomal gains on chromosomes 1, 2, 8, 12, 13, and 18. No definitive evidence of chromosome 14 haploinsufficiency was detected. Analysis of the X chromosome revealed no clear alteration at the FOXR2 locus (Xp11.21).

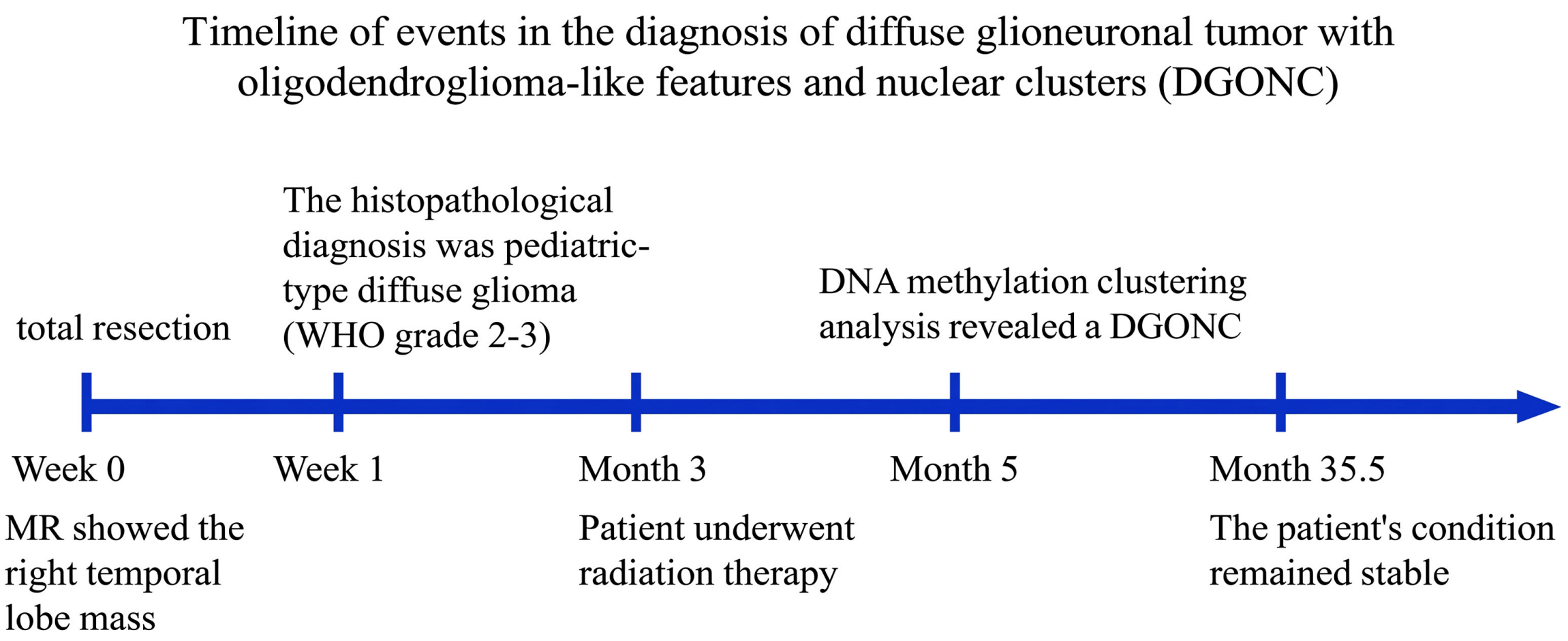

Supplementary Figure 3. Timeline of events in the diagnosis of DGONC. The timeline illustrates the clinical course from initial presentation to long-term follow-up. At week 0, MRI imaging revealed a right temporal lobe mass, followed by total resection. Histopathological examination at week 1 established a preliminary diagnosis of pediatric-type diffuse glioma (WHO grade 2-3). The patient underwent radiation therapy at month 3. DNA methylation clustering analysis at month 5 revealed the definitive diagnosis of DGONC. At month 35.5 follow-up, the patient's condition remained stable.

© 2026 The Author(s). Published by Bentham Science Publisher.

This is an open access article distributed under the terms of the Creative Commons Attribution 4.0 International Public License (CC-BY 4.0), a copy of which is available at: https://creativecommons.org/licenses/by/4.0/legalcode. This license permits unrestricted use, distribution, and reproduction in any medium, provided the original author and source are credited.

HOW TO CITE:
Deng D, Fan C, Yang Y, Wang W, Zhao H, Gao S. MRI Features of Diffuse Glioneuronal Tumor with Oligodendroglioma-like Features and Nuclear Clusters: A Case Report and Literature Review. Curr Med Imaging, 2026; 22: e15734056482743.
http://dx.doi.org/10.2174/0115734056462689260813104254
